# Supplementary material for: Post-Harvest Atmospheric Pressure and Composition Modify the Concentration and Bioaccessibility of α- and β-Carotene in Carrots and Sweet Potatoes
Source: Foods. 2023 Nov 25;12(23):4262. doi: 10.3390/foods12234262 (PMC10706462; doi:10.3390/foods12234262)

Supplementary Figure S1. HPLC chromatograms.

(**a**) α-Carotene standard; **(b)**: β-Carotene standard; **(c)**: Sample.

a

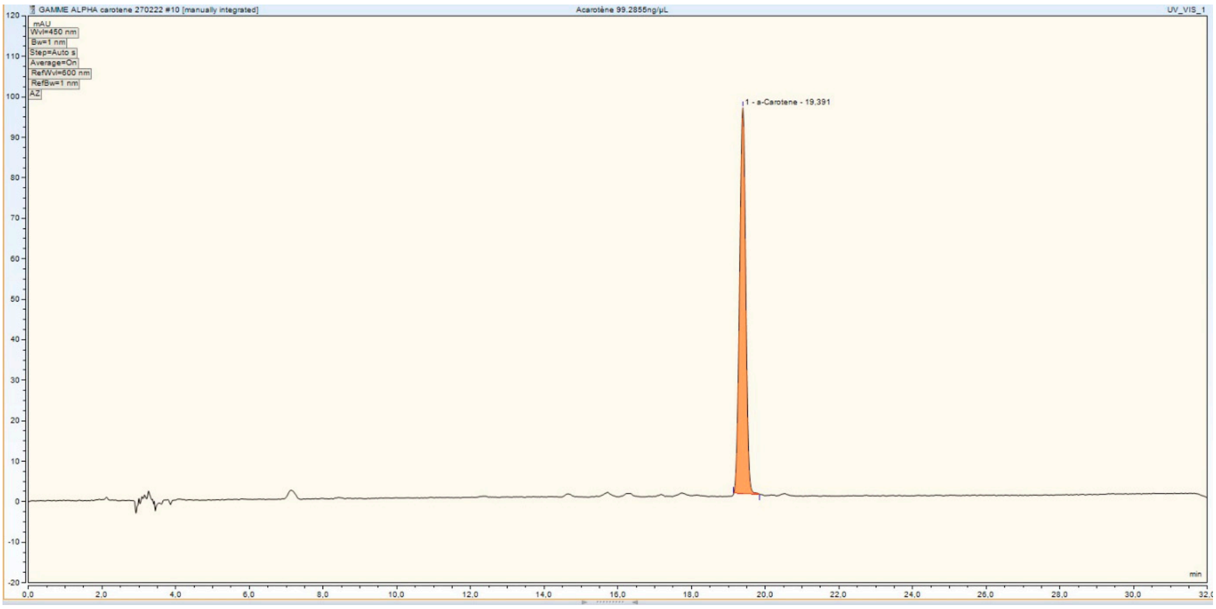

b

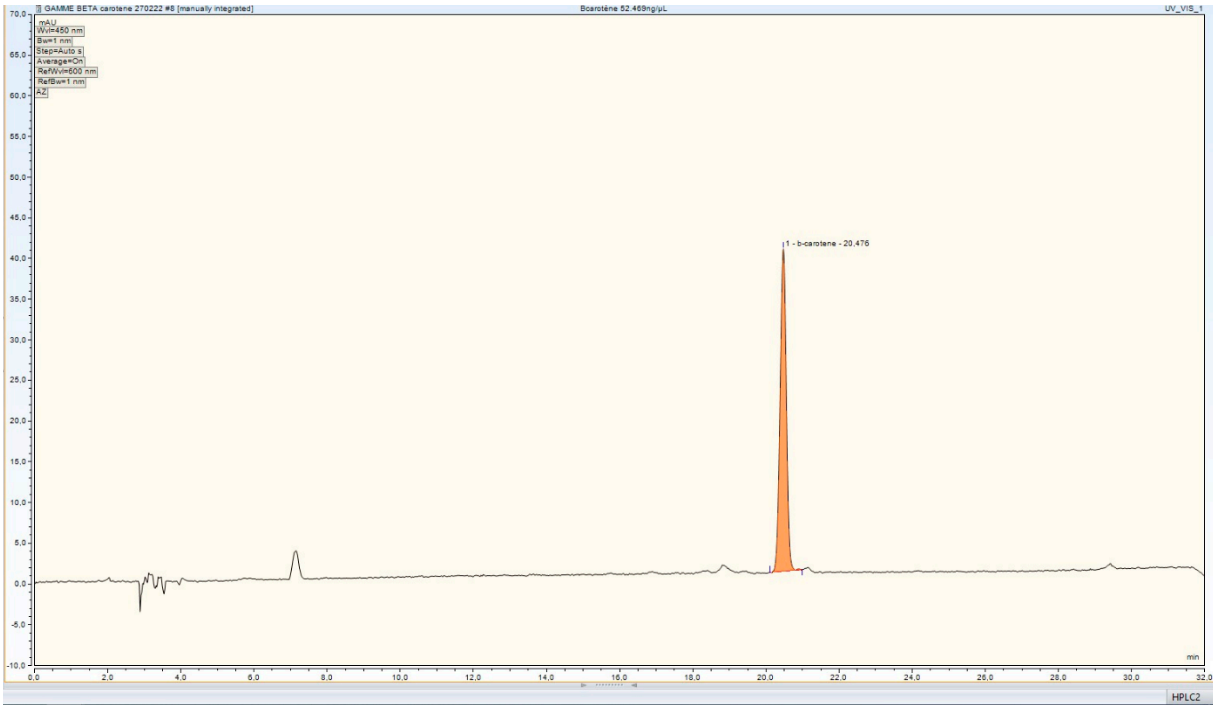

c

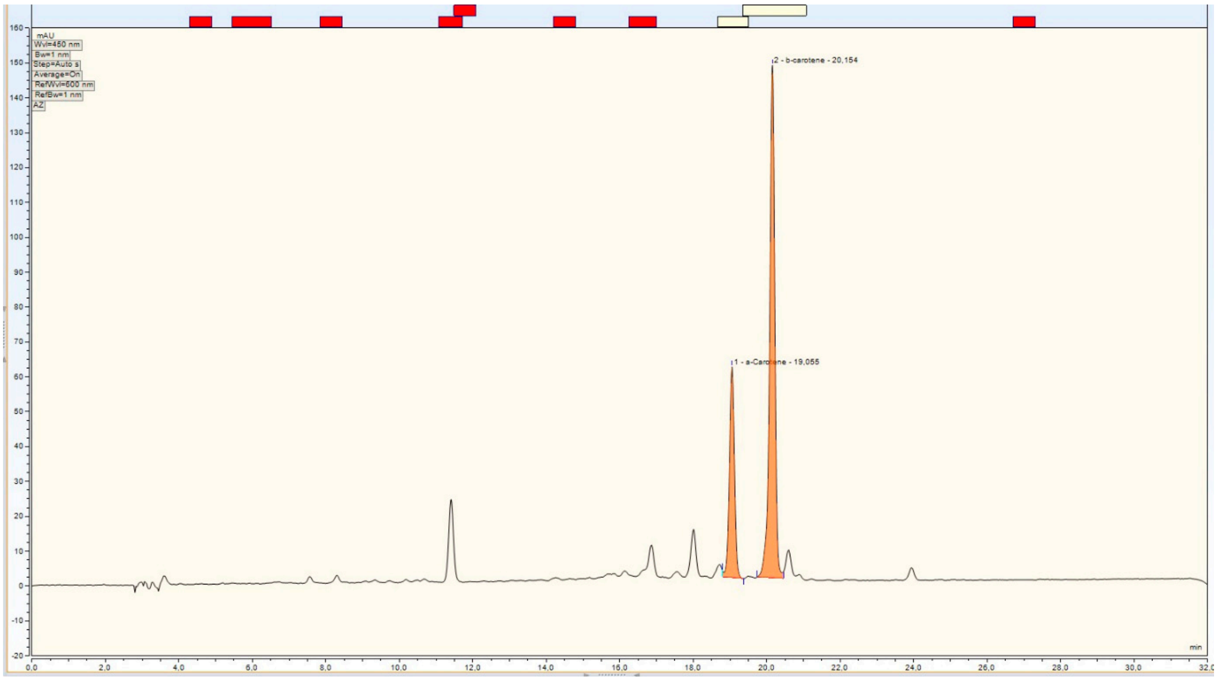
mAU
Wvl=450 nm
Bw=1 nm
Step=Auto s
Average=On
RefWvl=600 nm
RefBw=1 nm
AZ
2 - b-carotene - 20,154
1 - a-Carotene - 19,055
min

Supplementary Figure S2. (**a**) Concentration of *α*-carotene; **(b)**: Concentration of *β*-carotene in carrots stored at 20 °C for 10 days in modified atmospheric pressure and composition conditions. Control: P=1 bar, $P\mathrm{o}_2$=0.21 bar; normobaria/hypoxia: P=1 bar, $P\mathrm{o}_2$=0.03 bar; hypobaria/hypoxia: P=0.2 bar, $P\mathrm{o}_2$=0.04 bar; hyperbaria/anoxia: P=5 bar, $P\mathrm{o}_2$=0 bar. Values are means with their standard errors represented by vertical bars (n=5).

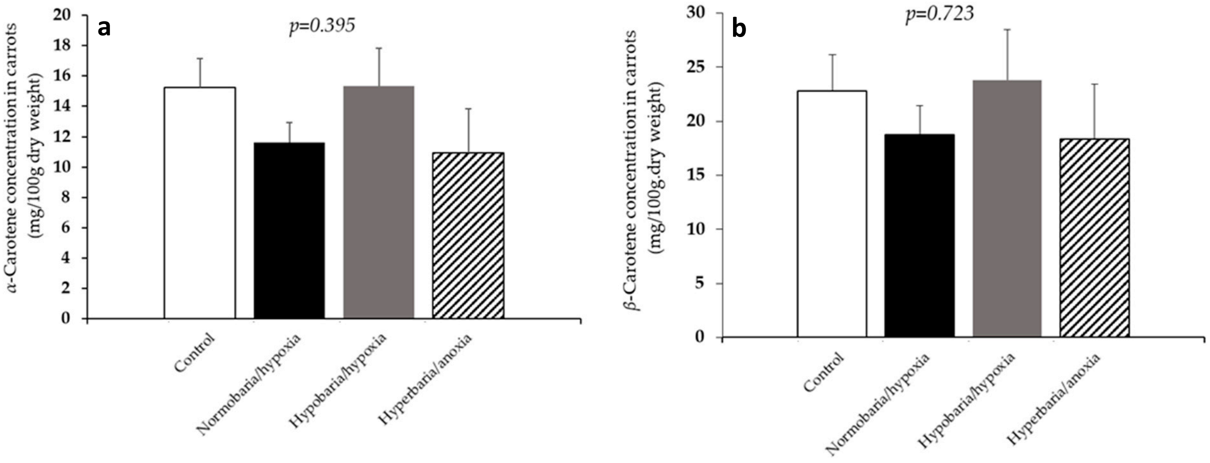

Supplementary Figure S3. (**a**) Concentration of *α*-carotene; **(b)**: Concentration of *β*-carotene in sweet potatoes stored at 20 °C for 10 days in modified atmospheric pressure and composition conditions. Control: P=1 bar, P_{O_2}=0.21 bar; normobaria/hypoxia: P=1 bar, P_{O_2}=0.04 bar; hypobaria/hypoxia: P=0.4 bar, P_{O_2}=0.02 bar; hyperbaria/anoxia: P=5 bar, P_{O_2}=0 bar, hyperbaria/hyperoxia: P=5 bar, P_{O2}=1.05 bar. Values are means with their standard errors represented by vertical bars (n=5).

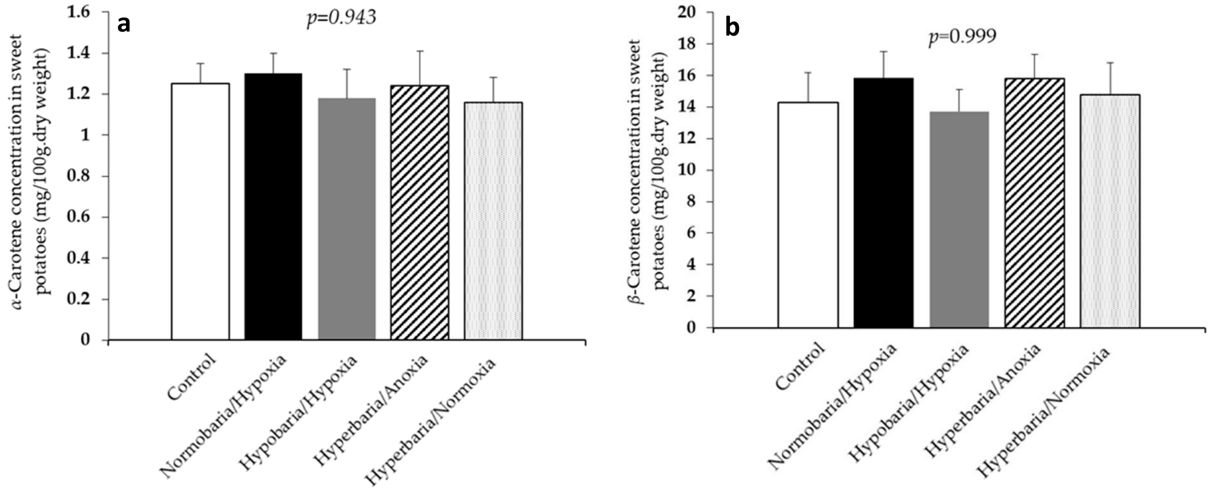

Supplementary Figure S4. (**a**) Concentration of *α*-carotene; **(b)**: Concentration of *β*-carotene in carrots stored at 20 °C for 10 days in modified atmospheric pressure and composition conditions. Control: P=1 bar, P_{O_2}=0.21 bar; normobaria/hypoxia: P=1 bar, P_{O_2}=0.03 bar; hypobaria/hypoxia: P=0.2 bar, P_{O_2}=0.04 bar; hyperbaria/anoxia: P=5 bar, P_{O_2}=0 bar. Values are means with their standard errors represented by vertical bars (n=5).

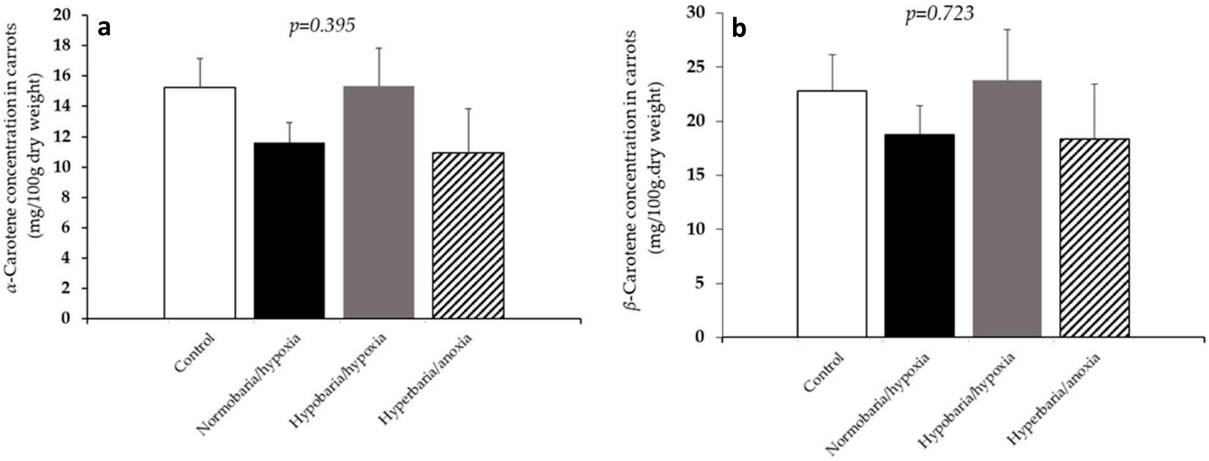

Supplement: Supplementary file 1 [file foods-12-04262-s001.zip › foods-2661186-supplementary.pdf]
